# Severe Worsening of Neuropathy Associated With Antibodies Against Myelin-Associated Glycoprotein by Rituximab Resembling Chronic Inflammatory Demyelinating Polyneuropathy

**DOI:** 10.7759/cureus.83338

**Published:** 2025-05-02

**Authors:** Johnny Dang, Yuebing Li

**Affiliations:** 1 Department of Neurology, Cleveland Clinic, Cleveland, USA

**Keywords:** chronic inflammatory demyelination polyneuropathy, demyelination, electromyography (emg), myelin associated glycoprotein, polyneuropathy

## Abstract

The presence of antibodies against myelin-associated glycoprotein (anti-MAG) is typically associated with distal acquired demyelinating symmetric neuropathy (DADS), usually presenting with slowly evolving sensory more than motor distal predominant polyneuropathy. On electrodiagnostic testing, DADS tends to show findings indicating distal predominant demyelination. Despite being a predominantly demyelinating neuropathy, DADS is resistant to multiple immunotherapies. Rituximab has been used as a successful treatment in a portion of patients with DADS, but rare cases of worsening of anti-MAG neuropathy have also been previously described. Here we describe a male patient with DADS who possessed high-titer anti-MAG antibodies. However, immediately following two doses of rituximab, he demonstrated a significant deterioration with acute proximal limb weakness and positive response to treatment of corticosteroid and intravenous immunoglobulin, resembling chronic inflammatory demyelinating polyneuropathy (CIDP).

## Introduction

Distal acquired demyelinating symmetric neuropathy (DADS) is typically a slowly progressive polyneuropathy manifesting with distal sensory loss, gait ataxia, and minimal distal weakness. Its electrodiagnostic features include distally predominant demyelination manifesting as significantly prolonged distal motor latencies without conduction block. DADS is resistant to multiple immunotherapies and can lead to significant disability over the course of several years [1]. Antibodies against myelin-associated glycoprotein (anti-MAG) have been implicated in DADS and recent studies with confocal microscopy have demonstrated colocalization of anti-MAG and C3d in areas of widely spaced myelin, suggesting a pathogenic role of the anti-MAG antibody in this entity [2].

Rituximab is a mouse-human chimeric monoclonal antibody against the CD20 antigen on B lymphocytes, inducing B lymphocyte depletion by a combination of the following mechanisms: complement-mediated cytotoxicity, antibody-directed cellular cytotoxicity and stimulation of apoptosis. Rituximab has been shown to be effective in some patients with anti-MAG neuropathy while significantly reducing the anti-MAG antibody titer in the majority of patients [3]. However, rare cases of worsening anti-MAG neuropathy have been described previously following rituximab treatment [4-7]. Here we describe a patient with anti-MAG neuropathy who showed acute worsening following rituximab treatment that resembles chronic inflammatory demyelinating polyneuropathy (CIDP). 

## Case presentation

A 59-year-old male presented with a two-year history of numbness and tingling in his toes that gradually progressed up along his legs, loss of balance, and slowly progressing leg weakness causing difficulty walking. There was a history of mild diabetes mellitus. Initial neurologic examination was notable for the presence of mild bilaterally symmetrical distal lower extremity weakness (big toe extension 3 based on Medical Research Council (MRC) scale and equivocal dorsiflexor weakness), global hyporeflexia, reduced pinprick sensation distal to the palms and knees, reduced vibration sense up to the bilateral knees, and absent proprioception of the bilateral toes. An electrodiagnostic study of the lower extremity demonstrated a sensorimotor polyneuropathy with absent bilateral sural and superficial peroneal sensory responses, absent bilateral tibial motor responses, and abnormal peroneal motor responses with increased distal motor latencies of 17.7 milliseconds (ms) on the left and 18.05 ms on the right (normal < 6 ms). Further workup revealed the presence of IgM lambda monoclonal protein, positive anti-MAG with a titer of 50,410 Bühlmann titre units (BTU) (normal <1000) (Table 1). Bone marrow biopsy was negative. A diagnosis of DADS and anti-MAG neuropathy was made. He was managed symptomatically with pregabalin and duloxetine.

**Table 1:** Key blood test results MAG: myelin-associated glycoprotein; BTU: Bühlmann titre units

Parameter (unit)	Patient result	Reference range
Hemoglobin A1C (%)	6.6	4.3-5.6
Vitamin B12 (pg/ml)	539	232-1245
Antinuclear antibody	negative	Negative
Anti-MAG antibody (BTU)	50,410	0-999
Immunofixation	IgM and lambda	Negative
M protein concentration (g/dl)	0.25	0.00

Two months after the initial office visit, the patient reported progressively worsening weakness in his lower extremities and falling. On repeat examination, patient had equivocal weakness in distal upper extremities but an otherwise stable exam. A repeat electrodiagnostic study demonstrated absent sensory responses of the right median and ulnar nerves, a reduced right radial sensory response, distal motor latency prolongation of the right median nerve at 13.05 ms (normal upper limit of 4 ms), and that of the right ulnar nerve at 5.15 ms (normal upper limit of 3.1 ms) as well as conduction slowing. No conduction block or temporal dispersion was observed. It was felt that his presentation was consistent with the previously diagnosed DADS and anti-MAG neuropathy. Physical therapy for one month failed to lead to significant improvement. Due to the clinical worsening, it was felt that rituximab treatment was indicated.

**Figure 1:**
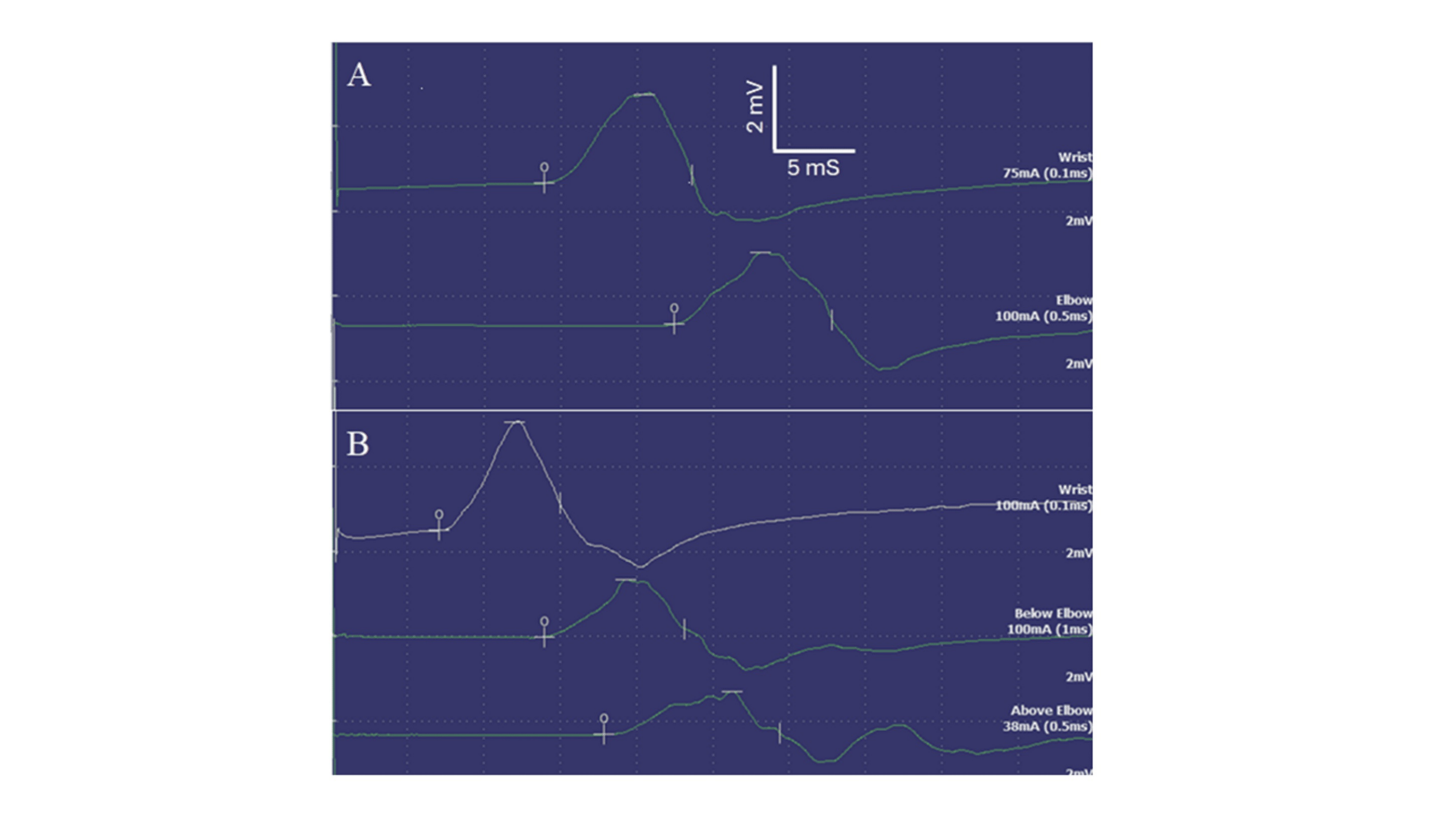
Results of motor nerve conduction study Results of motor nerve conduction studies indicate findings consistent with demyelination. A. Left median nerve stimulation at the wrist (top) and the elbow (bottom); B. Left ulnar nerve stimulation at the wrist (top), below elbow (middle) and above elbow (bottom). Prolonged distal latencies and slow conduction velocities are shown for both nerves. A 47% conduction block was observed for the left ulnar nerve between stimulations at the wrist and below elbow.

Three months following the initial office visit, the patient received two doses of intravenous rituximab at 375 mg per kilogram of body weight per meter square body surface area. Unfortunately, a quick clinical worsening of extremity weakness developed requiring the use of a walker or wheelchair to ambulate. Rituximab was discontinued, and he was admitted to the hospital for further evaluation. His muscle strength examination showed the following (MRC scale): elbow extension 4, finger abduction 4, hip flexion 3, knee flexion 3, knee extension 4, plantarflexion 3, and dorsiflexion 3. Magnetic resonance image of the lumbar spine with contrast was unremarkable. Repeat electrodiagnostic study revealed the appearance of partial conduction block and temporal dispersion in addition to the previously noted distal latency prolongation and conduction slowing (Figure 1). Needle examination of the proximal muscles of the lower extremity revealed the presence of neurogenic recruitment without significant changes on motor unit potential morphology. It was felt that the clinical and electrodiagnostic findings may have resembled a diagnosis of Guillain-Barré syndrome (GBS) or acute-onset CIDP. The patient was treated with intravenous methylprednisolone for three days followed by prednisone of 60 mg daily. He was also started on intravenous immunoglobulins (IVIG) with an initial loading dose of 2.0 grams then monthly maintenance of 1.0 gram per kilogram of body weight. Within three months following discharge, he was able to ambulate with a cane. At eight months post-discharge, he was able to ambulate independently. At month 10 post-discharge, his muscle strength examination was essentially normal. Repeat anti-MAG antibody testing showed a stable titer at 54,598 BTU.

## Discussion

Anti-MAG neuropathy typically presents with progressive distal sensory and motor deficits that evolve slowly over years or decades. Currently, there are no proven effective disease-modifying agents. Two randomized controlled trials examined the efficacy of rituximab in anti-MAG neuropathy and failed to demonstrate positive outcomes on the primary endpoints but rituximab was safe and well tolerated in both studies [8,9]. Despite negative results from controlled studies, rituximab remains the preferred off-label treatment option in many centers. Case series and retrospective studies indicated that approximately 30% to 50% of anti-MAG neuropathy patients may show positive responses to rituximab treatment [1,3].

A review of literature identified a total of 14 cases describing a worsening of anti-MAG neuropathy following rituximab administration [4-7,10-14]. Including our patient, nine were male and six were female. All worsening occurred acutely, ranging from one week to three months (median duration of two weeks) following rituximab initiation. In 10 patients, worsening of motor weakness was reported while worsening sensory symptoms and gait ataxia were noted in others. Thus, rituximab-associated worsening can be acute and severe in some while subacute and relatively mild in others. Changes on electrodiagnostic testing parameters such as worsening conduction slowing, amplitude reduction, and temporal dispersion were infrequently described [6,7]. In five patients, rituximab treatment was discontinued, which tended to result in improvement in several weeks. Additional immunotherapy including corticosteroid, IVIG or plasmapheresis was administered in seven patients which led to improvement in all patients. A total of 10 cases achieved complete recovery and five patients did not return to the baseline level of function.

The exact frequency of such worsening following rituximab is unknown without large-scale data. Sala et al. reported their observation of worsening in three patients (28%) among 11 patients with anti-MAG neuropathy treated with rituximab [7]. Another interesting observation was that the worsening by rituximab may not be reproducible on the same patient, as rituximab administration at an earlier or a later time may not cause similar worsening, indicating that the patient’s condition at that time may play a significant role in producing worsening [7].

Our patient showed a quick deterioration of proximal and distal limb strength, which was a sudden change from the slowly progressive course with minimal distal weakness. Therefore, this presentation does not appear to represent a worsening of the underlying slowly evolving neuropathy. The presence of features such as proximal muscle weakness, conduction block and temporal dispersion on electrodiagnostic testing, and gradual response to steroid and IVIG therapy over several months favored a CIDP-like presentation. In combination with prior reports of varying time courses of worsening, this rituximab-induced deterioration could represent a process similar to GBS or CIDP.

Our patient did not show a significant change in anti-MAG antibody level in association with the clinical worsening. This finding agrees with prior case reports. Among the 12 patients with reported change in anti-MAG antibody, only two showed significant titer elevation in association with worsening while the majority of others showed stable or declining antibody titers [6,11]. The underlying mechanism for rituximab worsening remains unclear, but it appears unrelated to a massive release of anti-MAG antibody in association with rituximab usage. In view of the significant improvement following immunotherapy in a portion of patients, a pro-inflammatory response induced by rituximab is likely.

## Conclusions

There is a need to be cautious about the use of rituximab in anti-MAG neuropathy. While rituximab treatment may provide benefit in some patients, the possibility of severe worsening of neuropathy manifestation needs to be considered and weighed. Rituximab-associated deterioration of anti-MAG neuropathy can be reversible with the use of immunotherapies, similar to those used in treating CIDP.
